# Challenges in HIV Vaccine Research for Treatment and Prevention

**DOI:** 10.3389/fimmu.2014.00417

**Published:** 2014-09-08

**Authors:** Barbara Ensoli, Aurelio Cafaro, Paolo Monini, Simone Marcotullio, Fabrizio Ensoli

**Affiliations:** ^1^National AIDS Center, Istituto Superiore di Sanità, Rome, Italy; ^2^Pathology and Microbiology, San Gallicano Institute, “Istituti Fisioterapici Ospitalieri”, Rome, Italy

**Keywords:** HIV-1 vaccine, therapeutics, HAART, functional cure, clinical studies, preclinical and clinical proof-of-concept studies

## Abstract

Many attempts have been made or are ongoing for HIV prevention and HIV cure. Many successes are in the list, particularly for HIV drugs, recently proposed also for prevention. However, no eradication of infection has been achieved so far with any drug. Further, a residual immune dysregulation associated to chronic immune activation and incomplete restoration of B and T cell subsets, together with HIV DNA persistence in reservoirs, are still unmet needs of the highly active antiretroviral therapy, causing novel “non-AIDS related” diseases that account for a higher risk of death even in virologically suppressed patients. These “ART unmet needs” represent a problem, which is expected to increase by ART roll out. Further, in countries such as South Africa, where six millions of individuals are infected, ART appears unable to contain the epidemics. Regretfully, all the attempts at developing a preventative vaccine have been largely disappointing. However, recent therapeutic immunization strategies have opened new avenues for HIV treatment, which might be exploitable also for preventative vaccine approaches. For example, immunization strategies aimed at targeting key viral products responsible of virus transmission, activation, and maintenance of virus reservoirs may intensify drug efficacy and lead to a functional cure providing new perspectives also for prevention and future virus eradication strategies. However, this approach imposes new challenges to the scientific community, vaccine developers, and regulatory bodies, such as the identification of novel immunological and virological biomarkers to assess efficacy end-points, taking advantage from the natural history of infection and exploiting lessons from former trials. This review will focus first on recent advancement of therapeutic strategies, then on the progresses made in preventative approaches, discussing concepts, and problems for the way ahead for the development of vaccines for HIV treatment and prevention.

## Introduction

The HIV epidemic represents one of the major health challenges worldwide, with important social and economical implications for public health. Approximately 34 million people are currently living with HIV, with a total of 24 million accumulated AIDS-related deaths, and 2.6 million new infections (1). With an estimated 6.1 million people living with HIV, South Africa’s epidemic remains the largest in the world. Worldwide, the pace of ART roll out to provide universal coverage is outpaced, especially in developing countries, by the number of new infections, rendering the objective almost impossible to achieve (2). Moreover, since ART alone cannot eliminate HIV-1 infection, the therapeutic regimen must be maintained for the lifetime, and this represents a major challenge for the patient (need of strict adherence, poor drug tolerability, drug interactions among antiretroviral agents, and other medications), which may lead to virologic failure and development of drug resistance, and an unbearable economical burden for the National Health Systems. Further, implementation of HAART therapeutic regimens requires a close clinical and laboratory monitoring and the commitment of large human and financial resources with an increasing economic burden for both developing as well as developed countries. This imposes major logistic obstacles to most developing countries, including an insufficient HIV testing, particularly in rural areas, the lack of infrastructures and socio-economical barriers (3–5). Finally, ART alone is unable to eliminate HIV-1 infection. In fact, there is evidence of persistent viral replication in compartments and reservoirs insensitive even to HAART (6). The discrete, though persistent, viral replication as well as HAART-resistant cell-to-cell virus transmission and homeostatic proliferation of infected memory T cells maintain the replenishment of HIV-1 reservoirs (7–16) ensuring virus persistence, while sustaining a residual immune dysregulation, which is associated to chronic immune activation, incomplete restoration of CD4 T cell counts, and lack of replenishment of central memory CD4 and CD8 T cells, which collectively represent the unmet therapeutic needs of ART. In turn, these unmet needs contribute to the residual disease and clinical complications. As a result, HIV-1 infection in the HAART era remains a chronic progressive disease and is now associated with novel syndromes, termed non-AIDS-associated diseases, including atherosclerosis, cardiovascular diseases, kidney and liver diseases, tumors, early aging, and drug-resistant co-infections (17, 18). These are life-threatening diseases reducing the quality of life of patients still experiencing a high-risk of hospitalization and death. The unmet needs of HAART have a heavy social impact and represent a considerable economic burden for National Health Systems.

Thus, novel, most effective therapeutic strategies are essential to allow a containment of the human, social, and financial resources necessary for the delivery of an effective health care against HIV/AIDS. Indeed, new approaches capable of targeting key pathogenic mechanisms of the virus life cycle, including the establishment and maintenance of virus reservoirs, are urgently needed to circumvent these problems and more effectively attack the virus, either as cART intensification or as an alternative to cART. To this end, novel drugs targeting additional steps in the virus life cycle, gene therapy approaches to render host cells resistant to infection, purging (“shock and kill”) strategies to empty viral reservoirs, as well as several different therapeutic immunization approaches are presently being investigated [reviewed in Ref. (19)].

## Therapeutic Immunization Strategies

In recent years, a growing attention has been given to the development of therapeutic HIV vaccines for treating people already infected with HIV-1. The development of an effective therapeutic vaccine might help at either intensifying ART efficacy, thus fulfilling the ART unmet needs, or, hopefully, at substituting the antiretroviral treatment (Table 1). The achievement of either objective might represent a considerable progress beyond the “state of art” of current therapeutic strategies against HIV infection, while ensuring a most favorable cost/efficacy ratio. In addition, an effective therapeutic vaccine may offer a promising alternative strategy to the recurrent failure of preventive HIV vaccines, based on the consideration that it can reduce HIV replication and transmission to healthy individuals. Moreover, therapeutic vaccination may apply to different intervention strategies according to its efficacy, ranging from cART intensification to drug simplification or, for the most effective ones, therapy interruption, or no cART initiation (see Tables 1 and 2). Thus, therapeutic vaccination has several advantages on the preventative counterpart, including a rapid and cost–effective proof-of-concept assessment of efficacy even in small phase I/II trials, the possibility to rapidly identify relevant biomarkers of protection, and it may be worth to develop even if not fully effective, since it may be used in association with antiretroviral drugs (see Tables 1 and 2). In fact, HIV therapeutic vaccines have been the topics of a conference recently held in Bethesda, MD, USA (20) underscoring the renewed and growing interest to pursue these types of immune interventions, whose potential and feasibility is becoming increasingly appreciated. However, a potential limitation of therapeutic vaccination as compared to the preventative one is that HIV-1 infected individuals may have a reduced immunocompetence, which may hamper both the elicitation and the strength of protective immune responses induced by the vaccine. As immune competence in HIV-1 infected individuals progressively declines over time, therapeutic vaccination early in the course on infection may be required to ensure best efficacy. As discussed later, this might also limit reservoir establishment and promote virus eradication.

**Table 1:** **Rationale for therapeutic immunization of HIV-infected individuals**.

**In HIV-infected Drug-Naïve individuals**
Delay or block of progression to AIDS or ARV Therapy
**In HIV-infected individuals in need of therapy or ARV-treated**
cART intensification to: a) Accelerate time-to-response to therapy b) Block or reduce virus transmission c) Help reduce reservoir size in patients given intensive ARV in acute infection d) Solve unmet needs of ART (immune activation, immune defects, and proviral DNA) Drug simplification Therapy interruption after ARV intensification

**Table 2:** **HIV/AIDS preventative and therapeutic vaccine … the continuum**.

Status	HIV-negative	Asymptomatic	At cART initiation	Upon cART initiation		
				Vaccine efficacy Low → High		
Goal	Prevention of infection	Block of progression (no cART need)	Faster and more efficient response to therapy	cART intensification	cART simplification	cART interruption
		(functional cure, eradication)				
Primary (short-term) end-points	HIV test Viral load CD4 counts	CD4 counts Viral load Time to cART initiation	CD4 counts Viral load Time to infection control	CD4 counts Viral load		
Secondary (long-term) end-points	None	Proviral DNA (Integrated, non-integrated, total) Neutralization of Tat/Env entry in DCs Markers of immune activation and reconstitution				

Although no therapeutic vaccine has been market approved, a growing number of vaccine candidates are being evaluated in phase I/II clinical trials conducted in both naïve and/or ART-treated patients [reviewed in Ref. (21) a comprehensive list of vaccine candidates available at: http://www.pipelinereport.org/sites/g/files/g575521/f/201407/Cure%20Immune%20Based%20and%20Gene%20Therapies.pdf]. Here, we briefly review the most representative of the diverse approaches undertaken.

InnaVirVax – (a spin-off of INSERM, Evry, France) is developing VAC-3S, a vaccine constructed to induce a humoral immune response against a highly conserved region of the envelope protein gp41 of HIV-1 known as 3S. The 3S domain has been shown to induce the expression of NKp44L on uninfected CD4 T cells, rendering them susceptible to lysis by NKp44^+^ activated NK cells, thus contributing to the massive T cell loss, which far exceeds the number of HIV-infected lymphocytes (22). Upon promising data in monkeys (23), it has recently been announced the start of a randomized, double-blind, placebo-controlled phase II study in 90 cART-suppressed adult subjects, with the primary endpoint being the induction of anti-3S antibodies, while overall tolerance and clinical safety, together with a comprehensive evaluation of the immunological end-points, inflammatory biomarkers, and vaccine’s immunogenic characteristics, represent the secondary end-points (ClinicalTrials.gov Identifier:NCT02041247).

Bionor Pharma (Oslo, Norway) is developing Vacc-4x, a peptide-based vaccine consisting of four synthetic peptides based on the HIV-1 p24 protein, injected multiple times intradermally together with GM-CSF. The results from a double-blind, randomized, phase II study conducted in 135 patients on effective cART showed that Vacc-4x is sae, immunogenic, and contributes to viral load reduction after cART interruption. However, the proportion of participants resuming cART before the end of the study and the CD4 T cell counts recorded during the treatment interruption showed no benefit of vaccination. In addition, it requires a long and complex vaccination schedule while providing a limited impact on viral load (24).

SEEK (London, UK) is developing PepTcell, a T cell epitope HIV vaccine consisting of synthetic peptides derived from the conserved regions of Vpr, Vif, Rev, and Nef. A randomized, placebo-controlled, dose finding Phase Ib/II trial was recently completed in 55 HIV-positive volunteers. After just a single subcutaneous injection, a significant reduction in viral load was observed in a minority of vaccinees who had received the high dose and had mounted both B and T cell responses to the vaccine (25).

FIT Biotech (Tampere, Finland) is developing GTU^®^ MultiHIV multi-gene, a vaccine based on six viral HIV proteins. The vaccine (clade B) has been tested in 60 untreated, asymptomatic, HIV-1 subtype C infected adults enrolled in a single-blinded, placebo-controlled Phase II trial in South Africa. The vaccine was safe and well tolerated. Significant declines in plasma HIV-RNA load and increases in CD4^+^ T cell counts were observed in the vaccine group compared to placebo, which were more pronounced after intramuscular (IM) administration (26).

Similarly, Profectus Biosciences (Baltimore, MD, USA) is developing TheraVax, a multi-antigen HIV vaccine in which a plasmid DNA encoding Env, Gag, Pol, Nef, Tat, and Vif is administered by electroporation, in combination with interleukin-12 plasmid DNA followed by a boost with same the antigens vectored by a recombinant vesicular stomatitis virus (rVSV) delivered intramuscularly. A randomized, double-blind, placebo-controlled phase I study in cART-treated patients (*n* = 50) has started in USA and results are expected by November 2016 (ClinicalTrials.gov Identifier: NCT01859325).

Genetic Immunity (Budapest, Hungary) is developing DermaVir, a DNA vaccine encoding 15 HIV proteins administrated by skin patches. A randomized, placebo-controlled, dose-ranging Phase II study in 36 HIV-infected individuals naïve to cART confirmed that the vaccine, administered three times, 6 weeks apart, is safe and immunogenic (27). A 0.5 Log_10_ reduction of plasma RNA copies per milliliter was seen in the arm immunized with the intermediate DermaVir dose (0.4 mg). However, no amelioration of CD4^+^ T cell counts was recorded (27).

GeoVax (Atlanta, GA, USA) is developing MVA/HIV62B, a two components’ vaccine: a recombinant DNA vaccine and a recombinant MVA (modified vaccinia Ankara) vaccine. Both produce non-infectious virus-like particles displaying HIV clade B Env, Gag, and Pol proteins. An open label phase I study in HIV-1 infected adults on successful ART who initiated therapy within 18 months from seroconversion (*n* = 9) is ongoing (ClinicalTrials.gov Identifier: NCT01378156).

Theravectys SAS (a spin-off of the Pasteur Institute, Paris, France) is developing THV01, a vaccine based on lentiviral vectors (01 and 02), both encoding HIV-1 Clade B Gag, Pol, and Nef proteins and exploiting the HIV DNA flap sequence, which permits the nuclear import of HIV in non-dividing cells, including dendritic cells (DCs), thus optimizing antigen immunogenicity. Upon promising efficacy data in macaques (28), Theravectys recently announced the initiation of a Phase I/II randomized, double-blind, placebo-controlled trial of 36 patients in 6 clinical centers aimed at assessing the safety and tolerance of the vaccine and measure the quality and intensity of the induced immune response, which, in perspective, should allow therapy interruption (ClinicalTrials.gov Identifier: NCT02054286).

Argos Therapeutics (Durham, NC, USA) is developing tailor-made vaccines in that autologous DCs, loaded *ex vivo* with RNA encoding four (Gag, Nef, Rev, and Vpr) of the patient’s own HIV antigens plus CD40L, are reinjected into the patient intradermally, four times, 4 weeks apart. Results from the Phase IIa (*n* = 24) study indicate delay of cART resumption in treated subjects, but no improvement of CD4^+^ T cell counts (29).

Of note, none of these vaccine candidates is aimed at intensifying HAART efficacy in order to attack the virus reservoirs and restore the immune homeostasis. To this aim, the Italian National AIDS Center is developing a vaccine based on the biologically active HIV Tat protein. Results from phase I preventative (ISS P-001, ClinicalTrials.gov identifier: NCT00529698) and therapeutic (ISS T-001, ClinicalTrials.gov identifier: NCT00505401) studies have indicated that the Tat vaccine is safe and immunogenic (30–32) and more recently results from a randomized phase II trial (ISS T-002) in virologically suppressed HAART-treated subjects (ClinicalTrials.gov NCT00751595) indicate that Tat vaccination exerts a positive impact on immune activation and T and B cell dysregulation [Ref. (33, 34) and Ensoli et al., manuscript submitted], confirming the role of Tat in the pathogenesis of the HAART unmet needs. Tat immunization induced a restoration of CD4^+^ and CD8^+^ T cell number and functional central memory T cell subsets, of B and NK cell number and a reduction of immune activation as compared to subjects under effective HAART (33). Of importance, Tat immunization induced a statistically significant reduction of blood HIV-1 DNA load [Ref. (34) and Ensoli et al., manuscript submitted]. Effects were greatest with Tat 30 μg, given three times at monthly intervals, and under Protease Inhibitors (PI)-based regimens, with a predicted 70% HIV-1 DNA decay after 3 years from vaccination and a half-life of 88 weeks. HIV-1 DNA decay was associated with anti-Tat Abs and neutralization of Tat-mediated entry of oligomeric Env in DCs, which predicted HIV-1 DNA decay. A phase II randomized, placebo-controlled clinical trial (ISS T-003, ClinicalTrials.gov Identifier: NCT01513135) of the Tat vaccine has just been completed in South Africa in 200 HAART-treated individuals and the results are expected by the end of 2014. Strategies are in development for phase III registrative trials.

More recently, also Biosantech SA – France (a spin-off of ANRS) started developing a vaccine based on a synthetic form of Tat derived from Tat Oyi, an attenuated clade B HIV field isolate. Based on results in monkeys (35), Biosantech recently announced the start of a therapeutic phase I study in 48 patients under cART. The strategy is aimed to therapy interruption.

As an alternative to therapeutic vaccines strategy, Sangamo BioSciences (Richmond, CA, USA), is developing SB-728-T, an *ex vivo* gene therapy approach by which CD4^+^ T cells drawn from HIV-infected patients are modified *ex vivo* to disrupt the CCR5 gene in autologous CD4^+^ T cells, expanded, and reinfused to the patient. Results from a phase I study indicate that both CCR5 alleles have to be disabled to make the treatment effective (36). However, the complexity and costs of this approach, together with serious safety issues represent a major disadvantage, rendering the immune therapy, even if it was effective, accessible only to a small fraction of the HIV population (37). Nevertheless, alternative strategies to render the patient’s cells resistant to infection are being developed and may turn out to be more feasible than at present (38–40).

Altogether, the above studies demonstrate that the HIV/AIDS therapeutic vaccine field is rapidly expanding and portraits a substantial variety of approaches, which differ sensibly in many aspects, the most relevant being the antigen chosen (unlike preventative vaccines, regulatory and accessory genes are frequently targeted; in some cases almost the entire HIV genome is targeted), and the delivery systems, which range from simple subcutaneous, intradermal, or intramuscular vaccine administration to reinfusion of autologous DCs loaded *ex vivo* with the selected antigen(s), or, for the gene therapy approaches, of genetically modified autologous target cells.

As compared to preventative approaches, the therapeutic setting provides the unique opportunity to evaluate vaccine efficacy in a more rapid and convenient manner, hopefully speeding up the identification and development of effective vaccine candidates. However, key clinical end-points and appropriate virological and immunological biomarkers to properly assess the therapeutic efficacy in more advanced trials still need to be established and agreed upon. To address these issues is a priority to grant advancement of therapeutic, and possibly preventative, vaccines.

## Preventative Vaccination Strategies

Despite almost 30 years of efforts, a preventative HIV vaccine is still lacking and only four types of HIV vaccines have been tested in six HIV vaccine efficacy trials so far (41, 42).

The first efficacy trials were conducted in high-risk populations immunized with a mixture of monomeric form of gp120 Env from two different clade B (VAX004) or from clade B and E (VAX003). Both vaccines, aimed at inducing neutralizing Abs (NAbs), failed to prevent infection (43, 44) and investigators turned their attention and hopes to vaccines aimed at inducing CD8 T cell responses.

The MRK rAd5 vaccine consisted of HIV-1 Gag, Pol, and Nef delivered by three different recombinant adenovirus 5 (rAd5) vectors and aimed at inducing protective CD8^+^ T cell responses. The phase IIb “test-of-concept” STEP trial (also termed HVTN 502 or Merck V520-023 study) was stopped due to evidence of enhanced risk of acquisition of infection, especially in those uncircumcised and with pre-existing antibodies to the vector. Because of this serious safety concern, the companion Phambili trial (HVTN 503) conducted in South Africa was also halted (45, 46). A very recent analysis confirmed that also in the Phambili trial the rate of acquisition of HIV infection was higher among vaccinees, especially during the long-term follow-up, for unknown reasons, although early unblinding due to trial stop might have influenced risk behavior (47). Of note, the temporal windows of increased risk of acquisition were the opposite in HVTN 502 (early after vaccination) and HVTN 503 (late after vaccination), underscoring the complexity of the factors involved, including differences in the risk populations targeted. A detailed analysis of these rAd5-based trials showed a strong activation of Ad5-specific (but not HIV-specific) CD4 T cells in the gut mucosa of vaccinees, which may explain their enhanced susceptibility to infection (48), a finding reproduced in the monkey model (49). Nevertheless, *post hoc* “sieve” analysis of breakthrough infections demonstrated that the vaccine had exerted some immune pressure, as indicated by the appearance of virus-escape mutants (50). However, the immune pressure was against HIV-1 epitopes contained predominantly in highly variable regions infrequently targeted in the course of the natural infection, domains which can presumably tolerate escape mutations (51). Thus, vaccines aimed at generating CD8^+^ T cell responses may still be a valid option, provided that better strategies and vectors are identified.

In fact, another rAd5-based preventative phase III trial was stopped in 2013 due to evident lack of efficacy (52). The HVTN 505 vaccine consisted of HIV-1 Gag, Pol, Nef, and three different Env from clade A, B, and C, delivered as DNA for priming followed by boosting with rAd5 vectors coding for all but Nef antigen. Despite these rAd5 vectors were less immunogenic than those used in the STEP trial and despite individuals seropositive for rAd5 were excluded from the trial, breakthrough infections were slightly higher among vaccinees as compared to placebo, casting serious doubts on whether to proceed further with adenoviral vectors (53) and, more in general, with vectors whose immune activating properties may exceed the capability to induce protective immune responses, thus favoring, rather than blocking, susceptibility to infection (48).

So far, only one efficacy trial has provided some evidence of low and transient (60% at 12 months but 31% at 42 months) protection from acquisition of infection. The RV144 Thai trial was an Env-based vaccine consisting of a priming with the CD4^+^ T cell-stimulating ALVAC canarypox expressing HIV-1 gag/pr/gp41/gp120 followed by the VAX004 gp120 Env of B and E clade, the very same used in the AIDSVAX trial reported above. Intriguingly, protection correlated with titers of IgG against the V1V2 loop of Env, which were not neutralizing but mediating ADCC, whereas, when present, high titers of IgA against the C1 domain of Env actually abolished the IgG effect by interfering with the IgG binding to V1V2 (54). Of note, mostly low to medium-risk individuals were enrolled in the RV144 Thai trial, and vaccine efficacy dropped to 3.7% when only high-risk individuals were considered (55). Nevertheless, this was considered an important step ahead in vaccine development and stemming from these results, several new trials have been designed to reproduce and possibly increase the efficacy (56).

It is worth considering that the Env-protein based vaccines tested so far in clinical trials have utilized gp120 Env monomers or a truncated form of gp160 (HVTN 505), although the spikes present on the infectious virion are constituted by trimers of gp160, which differ from monomeric gp120 Env in terms of antigenic properties and conformational epitopes displayed (57). This further emphasizes the concept that vaccine design should be more “pathogenesis-driven” in order to effectively target key virus molecules, a notion to carefully consider in future vaccine development. An oligomeric Env that closely resembles the native protein has been recently generated, which may represent a better immunogen and a useful tool to detect valuable anti-Env Abs (58).

**Figure 1:**
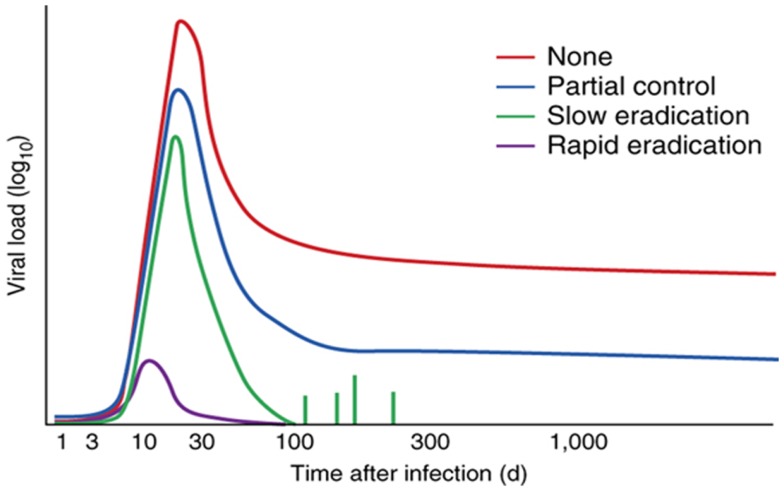
**Control of HIV-1 by vaccines that stimulate CTLs**. Effect of various T cell-stimulating vaccines (key) on viral load over time (with infection on day 0) during natural infection with HIV or SIV, showing the decrease in viral load achieved without a vaccine (none), by CTL responses [partial control; as in Ref. (92), for example], by the RhCMV vaccine (slow eradication) (60, 61) and by a hypothetical vaccine that targets the virus at the site of infection (rapid eradication). Reproduced with permission from Ref. (61).

On the other hand, approaches based on vaccines aimed at inducing protective CD8^+^ T cell responses only (HVTN 502 and 503) or in association to anti-Env antibodies (HVTN 505) have been largely disappointing. However, recent data obtained in the macaque model provided some encouraging results, although not necessarily easily transferable to human. This type of vaccine it is not expected to protect from infection acquisition, but rather to contain infection (i.e., low to undetectable plasma viral load and no CD4^+^ T cell loss), preventing progression to disease as well as virus transmission (Figure 1).

Strong control and apparent clearance of infection upon mucosal challenge with the pathogenic SIVmac239 was obtained with a replication competent simian cytomegalovirus (CMV) vector engineered to express SIV Gag, Tat, Rev, Nef, Env, and Pol (59–61). This strategy induced effector memory CD8 T cells which localized in peripheral tissues, including the mucosal portal of entry of the virus, thus providing the opportunity for the CTLs to attack the virus prior to dissemination. In fact, protected macaques experienced a reduced peak viremia, which rapidly subsided to undetectable levels, no CD4 T cell loss, no seroconversion, poor establishment of virus reservoirs in lymphoid tissues and effector tissues, eventually disappearing, a finding consistent with eradication of the infection (59–61). Protection appeared to be mediated by effector T cells present at the site of infection, although they were able to protect only 50% of the vaccinated monkeys, with the other half experiencing an infection comparable to controls (59–61). Intriguingly, CD8 T cells the effectors were restricted prevalently by class II rather than class I MHC antigens, and responses were very broad and persistent, features that appear to be peculiar to the replication competent modified CMV vector used (62). Besides underscoring the importance of the vector in the response to a vaccine, which also poses safety issues that will have to be exhaustively addressed before testing in human, this strategy provides a further indication that, to be effective, T cell responses have to be already in place at the portal of entry when the virus attacks. Still, if this defense line is overcome, the infection proceeds unaffected, indicating that effector T cells are necessary but not always sufficient to afford protection.

**Figure 2:**
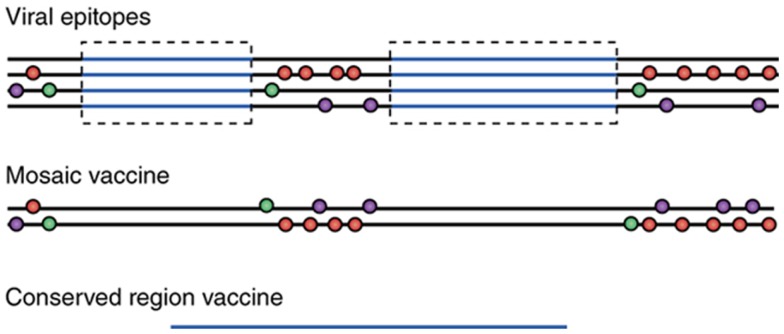
**Vaccines that deal with HIV-1 variability**. Construction of vaccines based on viral sequences in four viral isolates (top; simplified representation): horizontal lines indicate viral sequences; circles indicate sites of greatest variability between isolates (and potential escape mutations from CTL pressure; there may be more than two alternative sequences at each spot); and blue lines indicate regions of relative conservation (although in reality no region of HIV-1 is invariant). The mosaic vaccine (middle) is constructed to include the most common variants from the isolates in as few strands as possible while conserving naturally occurring sequence stretches. In the conserved region-containing vaccine (bottom), the relatively conserved regions (blue) are excised and then are “stitched” together (which creates an unnatural junctional region). The regions typically vary from 30 to 120 amino acids in length. Reproduced with permission from Ref. (63).

While the above strategy was aimed at inducing a specific type of effector cell, others are focusing on the selection of relevant epitopes [reviewed in Ref. (63)]. Mosaic antigens (64) and conserved antigens (65, 66) represent two potential strategies to address the challenges of global HIV-1 diversity (Figure 2). The first takes advantage of bioinformatics to generate mosaic antigen to cover most of the variants of each epitope displayed by circulating viruses with the aim of increasing the breadth of humoral and cellular immune responses, whereas conserved antigens aim to focus cellular immune responses on the most conserved viral sequences. Although the mosaic antigen approach seems more promising and has shown some efficacy in preclinical models (67), both strategies needs major improvements (better targeting of relevant epitopes, superior immunogenicity, durable immunity, and identification of correlates of protection) prior to progress to clinical trials.

**Figure 3:**
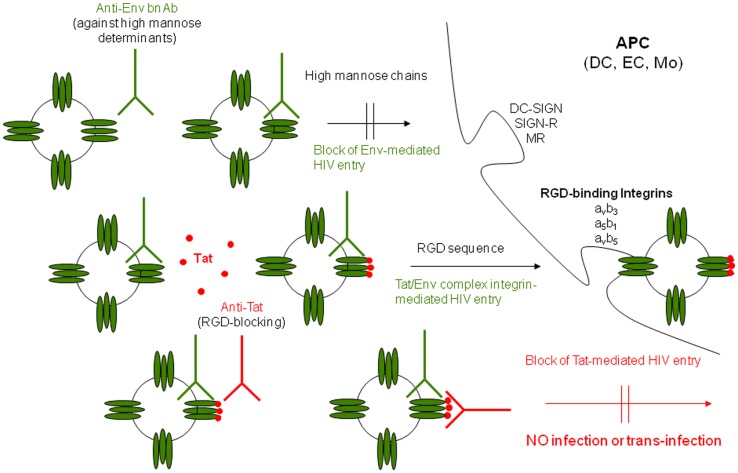
**Outcome of DC infection in the absence or presence of Tat, anti-Env, and/or anti-Tat antibodies**. Tat redirects HIV to RGD-binding integrins evading neutralization by anti-Env Abs and both anti-Env and anti-Tat Abs are required to block infection. Extracellular Tat released by infected neighbor cells binds to trimeric Env on HIV, decreasing recognition of C-type lectin receptors and promoting engagement of RGD-binding integrins, which are expressed by inflammatory DCs, macrophages, and endothelial cells (ECs) present at the site of infection. As a result, virions escape neutralization by anti-Env Abs directed against high mannose determinants and enters target cells upon binding to RGD-binding integrins. Anti-Tat Abs neutralize this binding, preventing virus entry through RGD-binding integrins. DC-SIGN: dendritic cell-specific intercellular adhesion molecule-3-grabbing non-integrin; SIGN-R: homolog of DC-SIGN present on ECs; MR: mannose receptor [modified from Ref. (68)].

A further approach based on a “pathogenetic” assumption, and thus aimed at inducing effective immune responses against a key HIV virulence factor, has been developed by targeting the HIV Tat protein. Tat vaccination represents an example of a “pathogenic-driven” intervention potentially effective for both preventative and therapeutic objectives, since it is aimed at blocking virus transmission/spreading. The rationale is based on the evidence that HIV-1 Tat, which is necessary for HIV gene expression, replication, and cell-to-cell transmission, appears also to be critical in the initial steps of virus acquisition. In fact, it has been recently shown that Tat, which is present on virus particles, binds to Env spikes promoting HIV infection of DCs and spreading to T lymphocytes even in the presence of anti-Env NAbs and that anti-Tat Abs are necessary to restore neutralization (Figure 3) (68). This evidence provides some explanation to the repeated failure of preventative vaccines based solely on Env and indicates that Tat may represent an optimal target for preventative interventions either alone or in combination with oligomeric Env.

Preclinical studies in cynomolgus monkeys have shown that immunization with a biologically active Tat protein or *tat* DNA is safe, elicits a broad and specific immune response and, most importantly, induces a long-term protection against infection with a highly pathogenic SHIV-89.6P encoding Tat of HIV-1, which rapidly causes AIDS and death in these monkeys (69, 70). Both Tat protein and *tat* DNA elicited memory Tat-specific Abs, CD4^+^, and CD8^+^ T cell responses in protected monkeys, which did not show signs of systemic infection throughout a 104-week follow-up or even upon a further boosting with tetanus toxoid (71), providing clear evidence of long-term containment of virus replication and spread in blood and tissues (72). A retrospective analysis of 112 Mauritian cynomolgus macaques from different preclinical trials, vaccinated (*n* = 67) or not (*n* = 45) with Tat and challenged with the SHIV-89.6P, showed that vaccination induced a significant reduction of the rate of infection acquisition at 10 MID_50_ (*P* < 0.0001), and contained acute CD4^+^ T cell loss at 15 MID_50_ (*P* = 0.0099). Of importance, vaccination also contained CD4^+^ T cell depletion (*P* = 0.0391) during chronic infection, irrespective of the challenge dose (73).

In a different approach, rhesus macaques primed mucosally with two replicating adenoviruses expressing HIV-1 Env and Tat, respectively, and boosted with the Tat and Env proteins became all infected following high dose intravenous SHIV-89.6P challenge. However, the Tat/Env vaccinated monkeys reduced chronic viremia by four logs as compared to controls (*P* < 0.0001). Of note, control of infection correlated with Tat and Env binding Abs (74).

Protection or containment of infection were also observed in cynomolgus macaques co-immunized with HIV-1 Tat and Env proteins and challenged intrarectally with a high dose (70 MID_50_) of the R5-tropic SHIV_SF162P4cy_ (68). In this case, the macaques had been primed twice intranasally with HIV-1 Tat and Env, given together with the LT-K63 mucosal adjuvant, and then boosted subcutaneously with Tat plus Env in Alum. No infection or a statistically significant reduction of viral loads and proviral DNA were observed in the vaccinated monkeys as compared to controls. Notably, proviral load in the inguinal lymph nodes was significantly lower in vaccinated monkeys as compared to controls, whereas it did not differ significantly in rectal biopsies, strongly suggesting the block of virus dissemination from the portal of entry (68).

In a further approach, the sterilizing immunity or control of infection observed in rhesus macaques immunized with a multi-component vaccine (multimeric HIV-1 gp160, HIV-1 Tat, and SIV Gag-Pol particles) delivered either systemically or mucosally and challenged orally or intrarectally with the C clade r5-tropic SHIV-1157ip correlated only with anti-Tat Abs against the N-terminus of Tat, as determined by a novel biopanning strategy which, using recombinant phages encoding random peptide libraries, allows a complete and unbiased profiling of the antibody repertoire and identification of epitopes associated with vaccine protection (75).

These studies strongly suggest that the induction of anti-Tat Abs may be key to achieve protective immunity against HIV/AIDS. In this regard is worth to note that in natural HIV infection, anti-Tat Abs are produced by only a small fraction of individuals (76, 77), while, in contrast, high Ab titers are produced against all other viral products (78). The reason for such a limited anti-Tat Ab response is unclear. However, when present, anti-Tat Abs correlate with the asymptomatic state and lower or no disease progression (79–83). In particular, a cross-sectional and longitudinal study, on 252 HIV-1 seroconverters, with a median follow-up time of 7.2 years, indicated that the presence of anti-Tat Abs is predictive of a slower progression to AIDS or immunodeficiency (83). Progression was faster in persistently anti-Tat Ab-negative than in transiently anti-Tat Ab-positive subjects, whereas no progression was observed in individuals persistently anti-Tat-Ab positive (83). Thus, anti-Tat Abs may have a protective role and represent a predictive biomarker of slower progression to AIDS.

The effects of anti-Tat Abs on the immunological, virological, and clinical outcome of HIV-infected subjects were recently assessed in a prospective observational study (ISS OBS T-003, ClinicalTrials.gov NCT01029548) conducted in asymptomatic drug-naïve HIV-infected adult volunteers (84). A significant association between the presence of anti-Tat Abs and a slower disease progression was found. In particular, anti-Tat Ab-positive patients showed a remarkable preservation of CD4^+^ T cells and containment of viral load for the entire follow-up (3 years), and no individuals with high levels of anti-Tat Abs initiated HAART during follow-up (84). Of note, the association of increasing anti-Env IgG titers with a lower risk of starting HAART occurred only in the presence of anti-Tat Abs, suggesting that anti-Tat and anti-Env Abs combined have increased HIV neutralizing effects by blocking the Tat/Env complex formation and virus entry, as shown earlier both *in vitro* and *in vivo* (68). Thus, both anti-Tat and anti-Env Abs appear to be required to efficiently block HIV disease progression. In contrast, anti-Env or anti-Gag Ab titers had no significant effects on CD4^+^ T cell counts and viral load in patients naïve to therapy with or without anti-Tat Abs (84).

Of importance, the results of a phase I safety and immunogenicity clinical trial (ISS P-001, ClinicalTrials.gov identifier: NCT00529698), indicate that Tat immunization is safe in HIV-negative healthy individuals and highly immunogenic, as it induced high titers of cross-clade, neutralizing anti-Tat Abs [(30–32), and unpublished results]. More recently a phase I, open label trial was conducted to assess the safety and immunogenicity in HIV uninfected healthy adult volunteers of a preventative vaccine based on the association of recombinant HIV-1 biologically active Tat and oligomeric Env deleted in the V2 region proteins (ISS P-002, ClinicalTrials.gov identifier: NCT01441193), which is currently under analysis.

## Discussion and Perspectives

An innovative targeting for HIV vaccine development should exploit lessons from former trials and pathogenetic mechanisms, taking advantage of the natural history of infection in humans (epidemiology, spontaneous control of infection). In fact, they provide valuable information: either the vaccine did not induce the responses desired and the immunization strategy has to be improved or changed, or it did it, but the responses were not protective, and the immunogen must be redesigned or the target antigen changed.

So far, HIV vaccine design based on structural knowledge has not been successful, nor have been empirical vaccines, reinforcing the concept that a rational pathogenetic approach must be undertaken to identify key virulence factors to be exploited for vaccine targeting. A “pathogenesis-driven” approach should be aimed at targeting key viral products responsible of virus transmission, activation and maintenance of virus reservoirs. Stemming from these considerations one may then argue whether a preventative vaccine should actually be different from a therapeutic one: a preventative vaccine capable of blocking virus entry and transmission should also be able to block virus spread within the infected host and, vice versa, a therapeutic vaccine targeting key steps of the virus life cycle and/or replication may rapidly control intra-host spreading after acquisition and hopefully eradicate the infection. In both cases, the rationale for vaccine design is to target key HIV virulence factor(s) required for virus entry/transmission and/or spreading. This is true in both healthy and already infected people, even those on suppressive HAART. In fact, in considering these two extremes, it appears that the viral dynamics is very similar. For infection acquisition, the virus must find/induce optimal conditions for the infection to occur, as indicated by the low transmission rate and the existence of individuals who remain uninfected despite being repeatedly exposed (85, 86). In fact, during the initial steps of viral infection the virus needs to overcome the mucosal barrier and to find proper target cells such as DCs, macrophages, activated CD4 T cells, to rapidly replicate and spread (87, 88). The presence of intraepithelial DCs, capable of sampling the “outside” and to rapidly bring and transfer the virus to neighbor CD4 T cells and activate them seems to be pivotal to infection establishment (87, 88). Accordingly, inflammation, immune activation and mucosal lesions (mostly due to other sexually transmitted infection, STI) enormously enhance sexual transmission (89, 90). From this first focus of infected cells and through discrete rounds of cryptic infection, virus establishes itself and initiates a productive infection. Strikingly, evidence of ongoing residual virus replication or reactivation have been reported even during suppressive HAART, which somewhat recapitulate and mimic the difficulties HIV encounters in the primary infection (91) Thus, understanding and blocking the mechanism(s) of virus transmission in primary as well as in chronic infection, in individuals either asymptomatic and naïve to drug or on HAART, will conceivably lead, respectively, to protection from infection and to virus eradication.

Thus, a “pathogenic-driven” strategy targeting a key virulence factor might be effective in both the preventative (healthy people) and the therapeutic approach (either in subjects naïve to drugs or on cART), by acting with the same mechanisms, in preventing/controlling HIV infection/progression to disease. The outstanding control and apparent eradication of infection conferred by CTL responses elicited upon preventative immunization with a CMV-vectored vaccine in a macaque model (58–60) is perhaps one of the best examples of a successful prevention obtained with a strategy aimed at inducing cellular responses (i.e., effector memory CTL) known to be associated to non-sterilizing immunity. Therefore, the distinction between preventative and therapeutic vaccine concepts and strategies should not necessarily be considered in terms of the development of different approaches, but rather in terms of targeting distinct populations (i.e., uninfected individuals vs. HIV-infected subjects) (Table 2). In considering this, it should be noted that the conduction of therapeutic trials may prove very useful and cost–effective in providing a first proof-of-concept of efficacy of a vaccine design and better define specific end-points and laboratory biomarkers to be assessed, before advancing to the very expensive and time-consuming preventative trials (Table 2). To these goals, novel immunological and virological biomarkers (in addition to viral load and CD4 T cell counts) should be taken into consideration in trial design in order to assess the achievement of efficacy end-points (i.e., assessment of functional T and B cell subsets, cellular and biochemical immune activation biomarkers, proviral DNA in reservoirs, cell-to-cell virus transmission, and virus neutralization). This approach imposes new challenges to the scientific community, vaccine developers and regulatory bodies, which require new paradigms and a new “way ahead.”

## Conclusion

New paradigms must be applied to develop efficacious preventative/therapeutic intervention strategies against HIV. These new concepts may also serve to combat epidemics by other agents. “Pathogenesis-driven” approaches should be considered with an open-minded attitude and should constitute the basis for a rationale vaccine design, taking also into account that structures do not always translate in immunogenicity and immunogenicity does not always translate in efficacy. The disappointing results from efficacy trials together with difficulties in translating preclinical studies to clinical trials, including, but not limited to, the uncertain predictivity of the results obtained in non-human primate models, also indicate that vaccine candidates should first be tested in infected individuals. This will provide a solid proof-of-concept to advance to the very expensive and time-consuming preventative trials. Further, new vaccine concepts and clinical trial designs should be considered and supported with proper funding. This also requires new methods for evaluation of projects where innovation is a key indicator. In fact, new vaccine design may require different end-points and biomarkers of efficacy as well as new testing for safety. Thus, regulatory bodies must be involved at an early stage of development and should be available to discuss the proper planning and conduction of innovative approaches.

## Conflict of Interest Statement

The Guest Associate Editor Marc H. V. Van Regenmortel declares that, despite having collaborated with the author Barbara Ensoli, the review process was handled objectively and no conflict of interest exists. The authors declare that the research was conducted in the absence of any commercial or financial relationships that could be construed as a potential conflict of interest.
